# Effect of Samarium Doping on the Energy Storage Properties of Bismuth Sodium Titanate-Based Lead-Free Ceramics

**DOI:** 10.1021/acsami.5c12016

**Published:** 2025-09-10

**Authors:** Xuyao Tang, Wanting Hu, Vladimir Koval, Jiangtao Zeng, Giuseppe Viola, Haixue Yan

**Affiliations:** † School of Engineering and Materials Science, 4617 Queen Mary University of London, Mile End Road, London E1 4NS, U.K.; ‡ Institute of Materials Research, Slovak Academy of Sciences, 04001 Kosice, Slovakia; § Shanghai Key Laboratory of Engineering Materials Application and Evaluation, 571917 Shanghai Research Institute of Materials, Shanghai 200437, P. R. China

**Keywords:** ferroelectric, relaxor, polar nanoregions, piezoelectric, energy storage, ceramics

## Abstract

Lead-free electroceramics have attracted significant research interest as alternatives to lead-containing systems due to concerns related to lead’s toxicity to human health and the environment. Solid solutions based on bismuth sodium titanate (BNT) and barium titanate (BT), particularly those with compositions near the morphotropic phase boundary (MPB), such as 0.94 Bi_0.5_Na_0.5_TiO_3_-0.06BaTiO_3_ (BNT-6BT), exhibit promising piezoelectric and ferroelectric properties. In this study, samarium (Sm) was introduced to partially replace both Bi and Na ions within the structure of BNT-6BT, at concentrations of 0.5 and 5 mol %, in samples labeled as BNTS0.5 and BNTS5, respectively. The addition of Sm modifies the A-site disorder on a nanometer scale, resulting in a decrease of the temperature *T*_s_ corresponding to a frequency-dependent shoulder in the dielectric permittivity and a significant increase of the temperature *T*_m_ corresponding to the maximum permittivity. Additionally, it was found that BNTS0.5 ceramic exhibits a relatively high piezoelectric coefficient (*d*_33_ = 164.7 pC N^-1^), while BNTS5 shows high recoverable energy density and energy storage efficiency (*W*_rec_ = 3.88 J cm^–3^ and η = 71.06%) at room temperature. With an exceptional recoverable energy storage intensity of 12.93 J V^–1^ cm^–2^ at room temperature, BNTS5 outperforms other similar materials, representing an excellent candidate for energy storage applications associated with the contribution of polar nanoregions. The two ceramics show significant potential for applications in piezoelectric energy conversion and energy storage devices.

## Graphical abstract

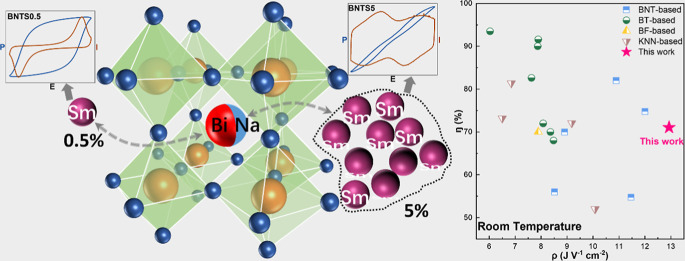

## 1 Introduction

In the last decades, lead-free ferroelectrics have attracted significant interest from the research community as alternatives to the widely used electroceramics containing lead, such as lead zirconate titanate (PZT).^1–4^ In 1991, Takenaka *et al*. carried out the first investigation on lead-free solid solutions combining bismuth sodium titanate (BNT) and barium titanate (BT), (1 – *x*)­Bi_0.5_Na_0.5_TiO_3_-*x*BaTiO_3_ (conventionally abbreviated as BNT-*x*BT).^5^ Electroceramics developed from ferroelectric solid-solution systems having compositions close to a morphotropic phase boundary (MPB) usually exhibit optimum piezoelectric and ferroelectric properties.^6,7^ In the BNT-*x*BT system, the MPB composition is located around *x* = 0.06–0.07 and is characterized by the coexistence of rhombohedral (R) and tetragonal (T) phases.^8^ Thus, materials derived from the 0.94Bi_0.5_Na_0.5_TiO_3_-0.06BaTiO_3_ (BNT-6BT) solid-solution with opportune modifications are expected to show improved properties for piezoelectric and ferroelectric applications.^9,10^ Moreover, their relaxor-like properties make them also suitable for electrical energy storage applications.^11–15^

The energy storage properties of a ferroelectric material, namely, the total energy storage density (*W*_tot_), recoverable storage energy density (*W*_rec_), and energy storage efficiency (η), can be estimated from the measured polarization–electric field (*P*–*E*) hysteresis loops by means of the following expressions:^16^

1 $$W_{tot}=\int_{0}^{P_{\max}}EdP$$

2 $$W_{rec}=\int_{P_{r}}^{P_{\max}}EdP$$

3 $$\eta =\frac{W_{rec}}{W_{tot}}\times 100\%$$

where *P*_max_ is the polarization at the maximum applied electric field (hereafter named maximum polarization), *P*_r_ is the remanent polarization, and *E* is the applied electric field. From these equations, one can deduce that an enhancement of the energy storage properties of a ferroelectric material can be achieved by an increase in the electrical breakdown strength and maximum polarization and a reduction of remanent polarization. Therefore, relaxor ferroelectrics could offer significant advantages for energy storage applications compared with conventional ferroelectrics.

BNT undergoes an electric field-induced transition from a relaxor state to a stable ferroelectric state, characterized by a large saturated polarization exceeding 35 μC cm^–2^.^5,11^ An introduction of Sr^2+^ ions on the A-sites of BNT has been reported to disrupt the long-range order of ferroelectric domains, leading to the formation of polar nanoregions and lower remanent polarization (*P*_r_).^17–19^ Similarly, the insertion of Zr^4+^ ions on the B-site was demonstrated to induce the transformation of microscopic domains into polar nanoregions and reduce *P*_r_.^20,21^ These chemical modifications ultimately led to improved energy storage properties.^17–21^

Previous studies on lead-based ferroelectric materials have shown that the A-site modifications with samarium (Sm) can enhance the piezoelectric performance.^22,23^ On the other hand, Sm-doping at specific concentrations was found to promote the formation of polar nanoregions, resulting in increased energy storage density.^22,24^ In the present study, the effect of Sm-doping on the piezoelectric and energy storage properties of lead-free BNT-6BT ceramics is investigated.

## 2 Experimental Section

Polycrystalline samples of the Sm-doped BNT-6BT system were prepared by a conventional solid-state reaction method. Samarium was introduced at two specific concentrations (0.5 mol % and 5 mol %) on the A-site of the perovskite ceramics with nominal compositions 0.94Bi_0.4973_Na_0.4919_Sm_0.0054_TiO_3_-0.06BaTiO_3_ (BNTS0.5) and 0.94Bi_0.4734_Na_0.4202_Sm_0.0532_TiO_3_-0.06BaTiO_3_ (BNTS5). For each composition, half of Sm was designed to partially substitute Bi, and the other half of Sm to partially replace Na. The specific amounts of Bi and Na replaced were determined based on the charge balance condition. For instance, in BNTS0.5, the amount of Sm^3+^ is 0.0054 moles. Since half of Sm^3+^ replaces Bi^3+^, the amount of the latter after doping is given by 0.5 – 0.0027 = 0.4973 moles. Instead, the amount of Na^+^ is given by 0.5 – 0.0027 × 3/1 = 0.4919 moles. At the same time, to keep charge balance, Na vacancies ([*V*′_Na_]) on the A-site were created, whose amount is given by: [*V*′_Na_] = 0.0027 × 2 = 0.0054. Hence, the defect-chemistry-derived chemical formula of BNTS0.5 can be written as follows: 0.94 Bi_0.4973_ [Sm_Bi_]_0.0027_Na_0.4919_[Sm^••^_Na_]_0.0027_[*V*′_Na_]_0.0054_TiO_3_-0.06BaTiO_3_ (BNTS0.5). Based on the same criteria, the other composition can be specified as: 0.94 Bi_0.4734_[Sm_Bi_]_0.0266_Na_0.4202_[Sm^••^_Na_]_0.0266_[*V*′_Na_]_0.0532_TiO_3_-0.06BaTiO_3_ (BNTS5).

For solid-state processing of the ceramics, the starting materials were Bi_2_O_3_ (purity ≥ 99.9%, Sigma-Aldrich), Sm_2_O_3_ (≥99.9%, Alfa Aesar), Na_2_CO_3_ (≥99.5%, Sigma-Aldrich), BaCO_3_ (≥99.8%, Alfa Aesar), and TiO_2_ (≥99.8%, Sigma-Aldrich). All powders were dried at 200 °C for 12 h, weighed according to the stoichiometric formulas, and ball milled at a speed of 200 rpm for 5 h in ethanol using a planetary mill (Pulverisette 5, Fritsch) with zirconia balls as milling media. After overnight drying in air, the mixture was first calcined at 800 °C for 2 h and subsequently at 900 °C for 4 h. The calcined mixture underwent an additional 5 h ball milling (200 rpm) in ethanol using a planetary mill and zirconia balls to reduce the particle size. During the last 10 min of this ball milling, a binder (5 wt % polyvinyl alcohol solution) was added to improve powder compaction. After drying, the calcined powder-binder mixture was cold-pressed into pellets 13 mm in diameter and 1 mm in thickness under a pressure of 200 MPa. The pressed pellets were annealed at 800 °C for 2 h to remove the binder. They were then covered with the original powder to compensate for element volatilization and sintered at 1150 °C for 4 h. The Archimedes’ method was employed to determine the density of the sintered ceramics. The density was found to be >97% of the theoretical density. Table S1 (in the Supporting Information) lists the values of the Archimedes' density along with the relative density values, which were calculated using the XRD refinement data.

The crystallographic structure of the sintered samples at room temperature was studied by X-ray diffraction (XRD) using Cu-Kα radiation (Panalytical CubiX3 X-ray diffractometer, Malvern Panalytical, Netherlands). Raman spectroscopy was conducted on polished samples using a Renishaw Raman microscope (Gloucestershire, UK) with 633 nm wavelength laser and a 20× microscope objective lens to focus and acquire the scattered light. Raman scattering data were collected in the range of 100–950 cm^–1^. The microstructure of the sintered ceramics was examined on fracture surfaces by scanning electron microscopy (SEM) with an FEI Inspect F microscope (Hillsboro, Oregon, USA). In addition, a semiquantitative elemental analysis was carried out using an energy-dispersive X-ray (EDX) spectrometer (Oxford Instruments, UK) attached to the SEM unit.

To investigate the dielectric, ferroelectric, and piezoelectric properties, silver paste (Sun Chemical S.A. Ltd., C2050926P2, Bath, UK) was applied to both sides of polished ceramic samples and then fired at 700 °C for 10 min to obtain uniform electrodes. The dielectric properties were tested at room temperature in the frequency range from 100 Hz to 10 MHz using an impedance analyzer (4294A, Agilent, Hyogo, Japan). All samples were poled at room temperature by applying a 5 kV mm^–1^ DC field for 10 min using a high-voltage power supply (a model 2807, Alpha Series II, Brandenburg, Germany). The piezoelectric coefficient (*d*_33_) was directly measured with a quasi-static *d*_33_ meter (ZJ-3B, Chinese Academy of Sciences, China).

The temperature dependencies of the relative dielectric permittivity and loss were collected from room temperature to 650 °C in the frequency range 1 kHz–1 MHz using an LCR meter (a model 4284A, Agilent, Hyogo, Japan) connected to a PC-controlled furnace. To explore the ferroelectric properties of the ceramics, a ferroelectric tester (NPL, Teddington, UK) was used to acquire the current–electric field (*I*–*E*) and polarization–electric field (*P*–*E*) hysteresis loops.^25–27^ The measurements were carried out in silicone oil at selected temperatures in the range 25–200 °C using triangular waveforms of different amplitudes at different frequency.

## 3 Results and Discussion

Figure 1 shows the fitted XRD patterns of BNTS0.5 and BNTS5 ceramics at room temperature. From Figure 1a, one can see that the BNTS0.5 ceramic adopts a perovskite structure with no secondary phases, suggesting the successful incorporation of the Sm ions in the A-sites of the perovskite structure. The Rietveld analysis of the diffractograms revealed that BNTS0.5 consists of a polar rhombohedral phase (space group, SG: *R*3*c*) and a weakly polar tetragonal phase (SG: *P*4*bm*). The dominating phase in the mixed-phase structure is the rhombohedral *R*3*c* phase, which contributes to the XRD profile by 81%. The lattice constants of the *R*3*c* phase were estimated as follows: *a* = *b* = 5.522 Å and *c* = 13.517 Å. The refined lattice parameters of the weakly polar *P*4*bm* phase are obtained as follows : *a* = *b* = 5.527 Å and *c* = 3.905 Å. Both BNTS0.5 and BNTS5 ceramics have a dense and uniform grain structure, as shown in the insets of Figure 1a and b. The EDX mapping of the chemical elements (Figures S1 and S2 in the Supporting Information) suggests a uniform distribution of all elements in both ceramics.

**Figure 1:**
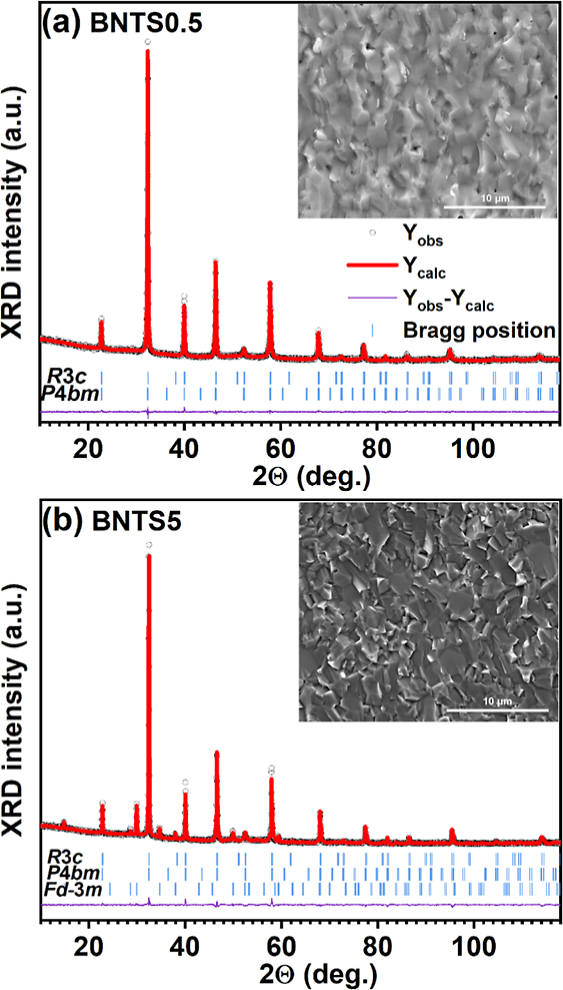
Fitted XRD patterns of (a) BNTS0.5 and (b) BNTS5 ceramics, as collected at room temperature. The insets show the SEM images of the respective ceramics.

The structural analysis of the BNTS5 ceramic (Figure 1b) revealed that, in addition to the rhombohedral *R*3*c* and tetragonal *P*4*bm* phases, the sample contains a pyrochlore Sm_2_Ti_2_O_7_-like impurity (ICDD PDF card no. 73-1699) characterized by a cubic symmetry (SG #227: *Fd*3̅*m*) with a lattice constant of about 10.33 Å. The presence of the secondary phase indicates that 5 mol % Sm doping is above the solubility limit. The pyrochloric impurity accounts for approximately 11% of the XRD pattern. It should be noted that the centrosymmetric secondary phase will have only a negligible impact on the ferroelectric properties of the main phase. Basically, it can indirectly decrease the measured polarization, dielectric permittivity, and piezoelectric coefficient, but its low permittivity is, on the other hand, useful to increase the dielectric breakdown strength, enabling high energy storage density.^15^ Detailed information about the structural refinements is given in Table 1.

**Table 1:** Refined Structure Parameters, Phase Fractions, and *R*-Factors for BNTS0.5 and BNTS5

sample	unit cell parameters (Å)	unit cell parameters (Å)	unit cell parameters (Å)	weight fraction (%)		*R*-factors and GOFs
	(Phase 1, *R*3*c*)	(Phase 2, *P*4*bm*)	(Phase 3, *Fd*3̅*m*)			
BNTS0.5	*a* = *b* = 5.521(2)	*a* = *b* = 5.527(1)		*R*3*c* = 81.00		*R_p_* = 9.230
	*c* = 13.517(2)	*c* = 3.905(1)		*P*4*bm* = 19.00		*R_wp_* = 5.430
	*α* = *β* = 90°, *γ* = 120°	*α* = *β* = *γ* = 90°				*R_exp_* = 3.070
	volume = 356.9(3) (Å^3^)	volume = 119.3(4) (Å^3^)				*χ* ^2^ = 3.140
BNTS5	*a* = *b* = 5.509(3)	*a* = *b* = 5.510(3)	*a* = *b* = *c* = 10.33(4)	*R*3*c* = 44.93		*R_p_* = 10.40
	*c* = 13.49(3)	*c* = 3.899(5)	*α* = *β* = *γ* = 90°	*P*4*bm* = 44.07		*R_wp_* = 5.870
	*α* = *β* = 90°, *γ* = 120°	*α* = *β* = *γ* = 90°	volume = 1102 (Å^3^)	*Fd*3̅*m* = 11.00		*R*_*exp*_ = 3.140
	volume = 354.6(5) (Å^3^)	volume = 118.4(2) (Å^3^)				*χ* ^2^ = 3.510

Note: GOF (*χ* ^2^) is the goodness of fit, *R*_*p*_ is the profile residual factor, *R*_*wp*_ is the weighted profile residual factor, and *R*_*exp*_ is the expected residual factor.

To further investigate the characteristics of the MPB structure, Raman spectroscopy has been employed.^11^ As shown in Figure 2, the Raman spectra of BNTS0.5 and BNTS5 ceramics are similar to those of other BNT-based ceramics.^28^ After a deconvolution of the spectra, the bands specifically associated with the rhombohedral and tetragonal structures were identified. The Raman active mode at ∼135.0 cm^–1^ can be assigned to A-site vibrations, whereas phonon modes above 200.0 cm^–1^ are associated with bending, stretching, and torsion of the TiO_6_ octahedra.^11,29,30^ The modes around 253.0 cm^–1^ (E­(TO) mode) and 526.0 cm^–1^ (B_1_ mode) belong to the rhombohedral phase, while those at around 312.5 cm^–1^ (B_1_/E (TO + LO) mode) and 600.0 cm^–1^ (A_1_(LO) mode) are characteristic of the tetragonal phase.^31–33^ The broad Raman feature near 800.0 cm^–1^ probably corresponds to the vibrations involving oxygen displacement in the TiO_6_ octahedra.^34^ The two distinct peaks at 536.5 cm^–1^ and 615.0 cm^–1^ in the Raman spectrum of BNTS0.5 tend to merge into a broad peak in the spectrum of BNTS5, suggesting an increased content of polar nanoregions compared to BNTS0.5.^35,36^

**Figure 2:**
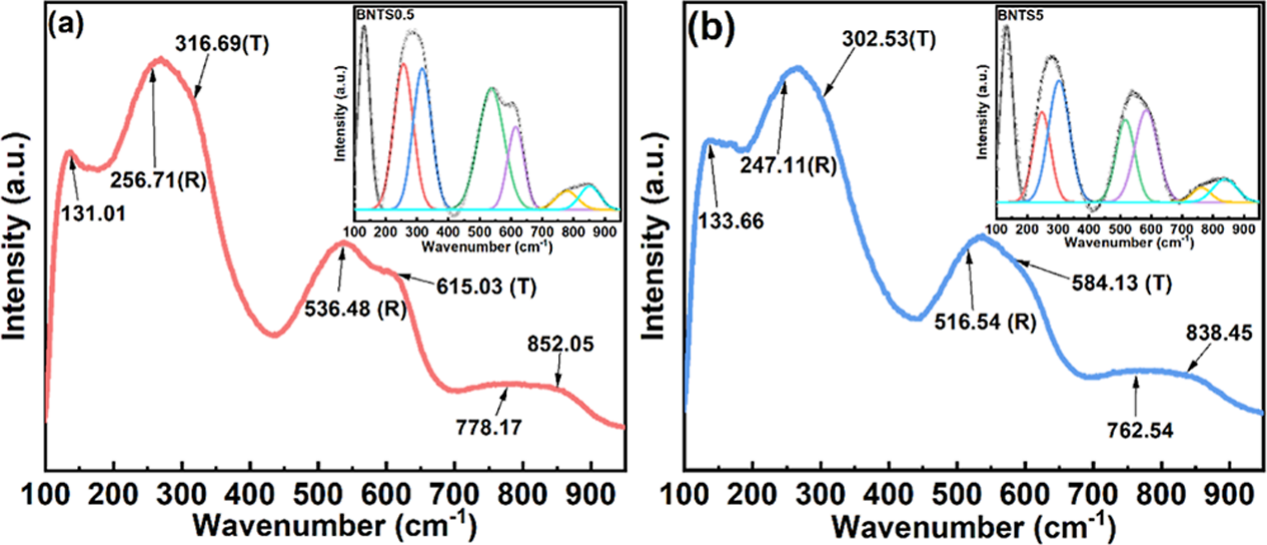
Raman spectra of (a) BNTS0.5 and (b) BNTS5 ceramics at room temperature (T—tetragonal phase, R—rhombohedral phase). The insets show Gaussian fit results after background removal.

The polarization–electric field (*P*–*E*) hysteresis loops and corresponding current–electric field (*I*–*E*) curves of the BNTS0.5 sample, as recorded under various electric field amplitudes at selected temperatures in the 25–200 °C range, are shown in Figure 3. At 25 °C and regime conditions (after the first electric field cycle), BNTS0.5 exhibits the characteristics of a classical ferroelectric material, with remnant polarization *P*_r_ = 28.01 μC cm^–2^ and coercive field *E*_c_ = 3.90 kV mm^–1^. After DC poling at room temperature, the ceramic showed a relatively high piezoelectric coefficient *d*_33_ = 164.7 pC N^-1^. When BNTS0.5 is heated to 50 °C, a clear reduction in the coercive field is observed (*E*_c_ = 3.20 kV mm^–1^) due to the additional contribution of the thermal energy to domain switching.^27^ At 75 °C, in contrast to the two peaks observed at room temperature, there are four peaks located at ± *E*_f_ and ± *E*_b_ in the *I*–*E* loop (Figure 3c, whereby the subscripts f and b stand for forward and backward, respectively). The current peaks at ±*E*_f_ are associated with the so-called “forward transition” from the weakly polar tetragonal phase to the polar *R*3*c* phase, taking place during electrical loading. The current peaks at ±*E*_b_ are related to the “backward transition”, occurring during electrical unloading or field reversal.^27^ It can be seen that the peaks at ±*E*_b_ are located close to *E* = 0, indicating that the backward transition at 75 °C begins during unloading and is completed during field reversal. As the temperature increases to 100 °C, the interval between the threshold fields −*E*_b_ (+*E*_b_) and +*E*_f_ (−*E*_f_) extends, leading to the reduced hysteresis of the polarization.^27^ Additionally, one can see that due to the reduced stability of the polar rhombohedral structure, the peaks at ±*E*_b_ appear during field unloading at elevated temperatures.

**Figure 3:**
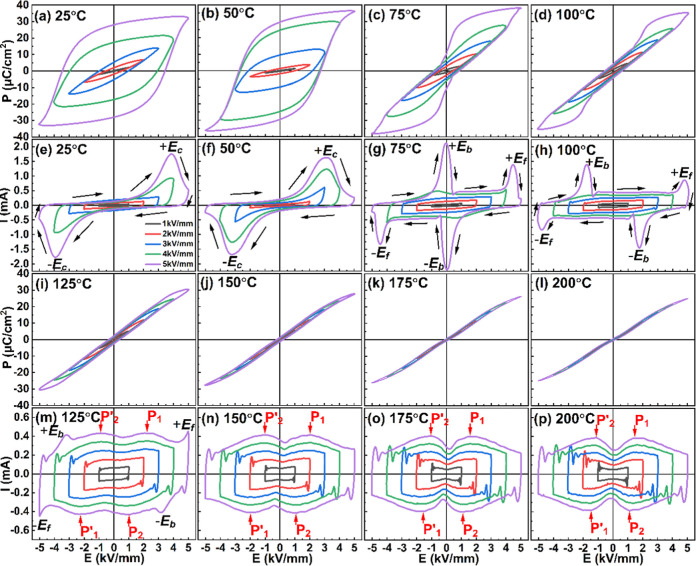
*P*–*E* (a–d and i–l) and *I*–*E* (e–h and m–p) loops of the BNTS0.5 ceramic, as recorded at eight different temperatures (25, 50, 75, 100, 125, 150, 175, and 200 °C) under different electric field amplitudes and 10 Hz frequency.

At 125 °C, the *I*–*E* loops show eight current peaks, four at ±*E*_b_ and ±*E*_f_ and the other four at the fields corresponding to P_1_, P_2_, P′_1_, and P′_2_, as shown in Figure 3m. Detailed information on the evolution of the *P*–*I*–*E* hysteresis loops in the temperature range 100–130 °C can be obtained from Figure S3 (in the Supporting Information), where ferroelectric data acquired from samples of the same composition are presented. According to earlier studies on similar BNT-based relaxor ferroelectrics,^37,38^ the peaks P_1_, P_2_, P′_1_, and P′_2_ can be attributed to mostly reversible “short-range polar state transitions”. At low electric fields, the field-induced transitions during electrical loading are manifested by the current peaks P_1_ and P′_1_ and at higher electric fields by the current peaks located at ±*E*_f_. Upon unloading, the material returns to its original state in two successive steps, first at ± *E*_b_ and then at a lower field corresponding to the current peaks P_2_ and P′_2_.

When the temperature reaches 150 °C, the current peaks at ±*E*_b_ and ±*E*_f_ disappear, and only the peaks P_1_, P_2_, P′_1_, and P′_2_ are observed (Figure 3n). From Figure 3n–p, it can be noticed that with increasing temperature, the difference between the electric fields corresponding to P_1_ and P_2_ peaks (Δ*E*) gradually decreases. The lower value of Δ*E* is reflected in the slim *P*–*E* hysteresis loops (Figure 3j–l), suggesting high energy storage efficiency (η).^17^ The *P*–*E* loops measured at 175 and 200 °C are notably slim, indicating relaxor-like behavior, which is typically associated with the presence of polar nanoregions at these elevated temperatures.^8,17^

Figure 4 shows the temperature dependencies of the relative dielectric permittivity (*ε_r_*) and loss (*tanδ*) of the unpoled and poled ceramics, as collected from room temperature to 650 °C at five different frequencies (1 kHz, 10 kHz, 100 kHz, 500 kHz, and 1 MHz).

**Figure 4:**
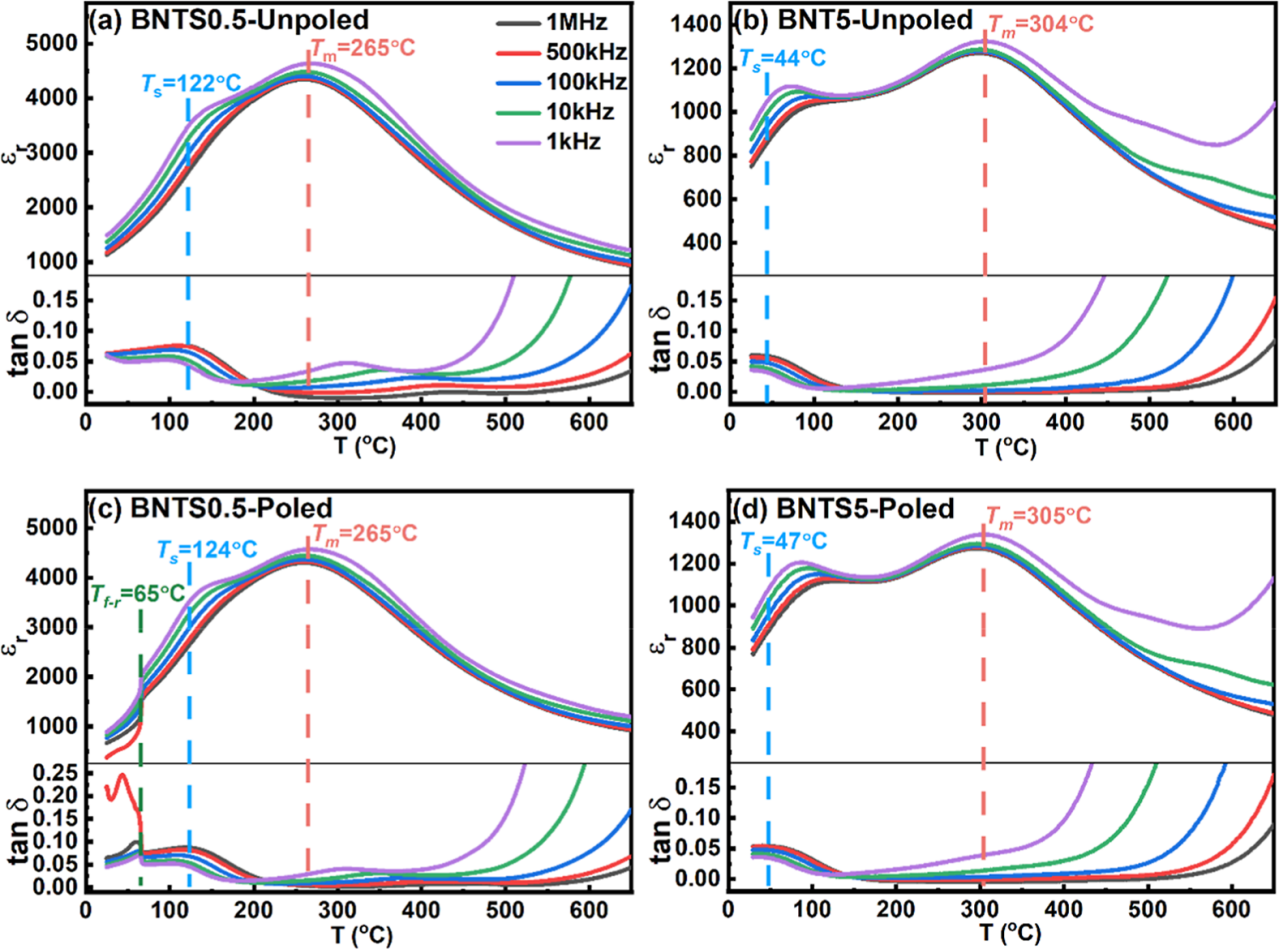
Temperature dependencies of the relative dielectric permittivity (*ε*_r_) and loss (*tanδ*) at five different frequencies for the unpoled and poled ceramics of BNTS0.5 (a,c), and BNTS5 (b,d).

It is widely known that the dielectric behavior of BNT-6BT ceramics is characterized by strong frequency dispersion and various anomalies in the temperature dependence of permittivity and loss.^16,39^ For the unpoled samples (Figure 4a,b), two main anomalies can be identified in the *ε_r_*/*tanδ* *vs* temperature curves. The first anomaly is manifested by the appearance of a distinct shoulder at the temperature *T*_s_, which has been determined from the hump in the *tanδ*–*T* plot.^39–41^ A pronounced dielectric relaxation phenomenon is evident near *T*_s_, and notably, the temperature at which tanδ reaches its maximum is positively correlated with the frequency increase. The second anomaly in the *ε_r_*–*T* plot is located at the temperature *T*_m_, which corresponds to the maximum permittivity temperature.

In the unpoled BNTS0.5 sample, *T*_s_ is around 122.0 °C and *T*_m_ is about 265.0 °C, while the unpoled BNTS5 ceramic has *T*_s_ close to 44.0 °C and *T*_m_ around 304.0 °C. For pure BNT-6BT ceramics, the *T*_s_ and *T*_m_ temperatures are about 124.0 and 242.0 °C, respectively.^35^ Apparently, the A-site substitution of Bi and Na ions by the Sm ions results in a lower *T*_s_ and higher *T*_m_ temperature. The features identified in the dielectric properties are also consistent with the observed hysteresis loops of the BNTS0.5 and BNTS5 ceramics. In BNTS0.5, the temperature *T*_s_ ∼ 122.0 °C can be correlated with the temperature where the *I*–*E* loop exhibits eight distinctive current peaks (∼125.0 °C). However, the BNTS5 sample does not show a higher number of peaks in the *I*–*E* curve (Figure 5) near its *T*_s_ ∼ 44.0 °C, suggesting that the sequence of the current peaks in the *I*–*E* loop strongly depends on the composition and temperature. At room temperature, BNTS0.5 finds itself into a nonergodic state and experiences an irreversible field-induced transition in the very first loading cycle; hence, at regime conditions (in the subsequent electric field cycles), the current peaks in the *I*–*E* loops resemble the typical patterns of ferroelectric materials, mainly reflecting domain switching. On the contrary, BNTS5 at room temperature shows an ergodic state and undergoes reversible field-induced transition; hence, the *I*–*E* loops usually show four visible current associated with forward and backward field-induced phase transitions. As shown in Figure 4, the dielectric behavior of the poled BNTS5 ceramic is rather similar to that of the unpoled ceramics, indicating the ergodic state of BNTS5. In contrast, BNTS0.5 shows some changes in the dielectric response after poling, which suggests the nonergodic state of the ceramic. More specifically, it exhibits a notable decrease in the frequency dispersion of *ε_r_* and *tanδ*, if compared to that of the unpoled state. In addition, a frequency-independent sharp anomaly in the permittivity and loss is observed at the temperature *T*_f*‑*r_ ∼ 65.0 °C, which corresponds to the transition from a ferroelectric to a relaxor state on heating.^11^ The anomalous thermal behavior of ε_r_ and tanδ at the measuring frequency of 500 kHz in the poled BNTS0.5 sample at and below *T*_f*‑*r_ (Figure 4c) can be attributed to the electromechanical resonance effect typically observed in poled ferroelectrics.^42,43^

**Figure 5:**
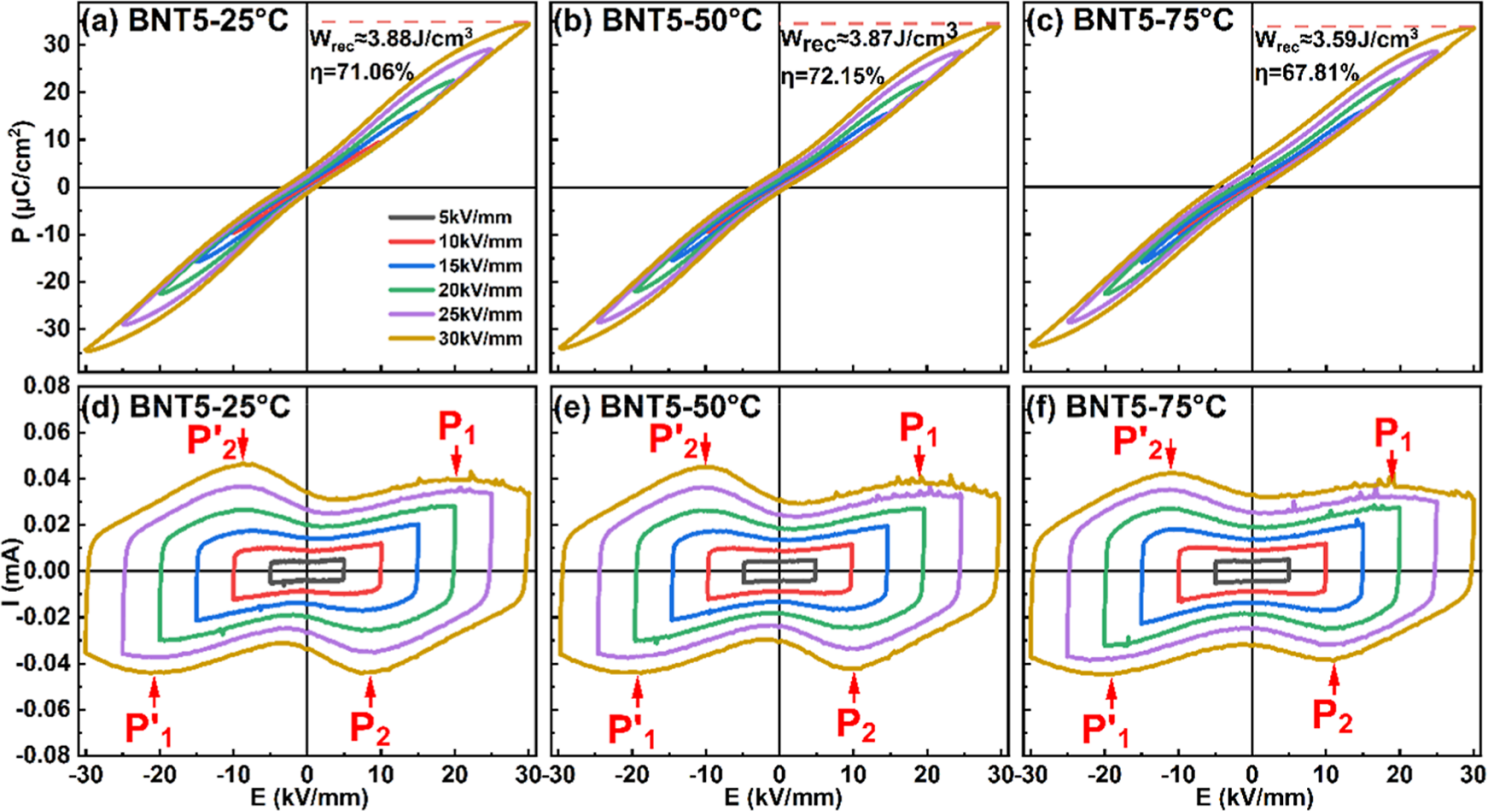
*P*–*E* (a–c) and *I*–*E* (d–f) loops of BNTS5 ceramics measured in the temperature range from 25 to 75 °C, at different electric field amplitudes from 5 kV mm^–1^ to 30 kV mm^–1^ at 10 Hz.

Figure 5 shows the *P*–*E* hysteresis loops and the corresponding *I*–*E* curves of BNTS5 ceramics at various electric field amplitudes at three different temperatures (25, 50, and 75 °C) around the temperature *T*_s_. The energy storage characteristics, namely, recoverable energy density (*W*_rec_) and energy storage efficiency (*η*) of the BNTS5 ceramic, were calculated from the recorded ferroelectric (*P*–*E*) data using eqs 2 and 3, respectively. At room temperature, BNTS5 exhibits a maximum *W*_rec_ of approximately 3.88 J cm^–3^ and an efficiency η of about 71.06% at an applied field of 30 kV mm^–1^, as a result of the low remanent polarization (*P*_r_ = 3.31 μC cm^–2^) and high maximum polarization (*P*_max_ = 34.27 μC cm^–2^). The reduced *P*_r_ value can be explained by the Sm-induced nanoscale disordering at the A-sites of the BNT structure. Sm^3+^ on the A site introduces heterovalent/heterosize disorder and a *V*′_Na_ vacancy to compensate charge imbalance. The defect Sm^3+^/*V*′_Na_ dipole pair produces a local random electric field and shortens the correlation length of polar nanoregions, resulting in lower *P*_r_. While BNTS0.5 was found to show a high piezoelectric coefficient due to high *P*_r_, the BNTS5 ceramic with low *P*_r_ at room temperature is more suitable for energy storage. By increasing the temperature to 50 °C, a slight decrease in *W*_rec_ to 3.87 J cm^–3^ occurs, while *η* significantly increases to 72.15%, resulting from an increased *P*_r_ (≈3.63 μC cm^–2^) and a reduction in maximum polarization (≈33.85 μC cm^–2^). As the temperature is increased to 75 °C, both *W*_rec_ and *η* decrease. The *P*–*E* hysteresis loops and the corresponding *I*–*E* curves of BNTS5 at 100 and 125 °C, and the temperature dependence of *W*_rec_ and *η* for BNTS5 are shown in Figures S4 and S5 in the Supporting Information.

**Figure 6:**
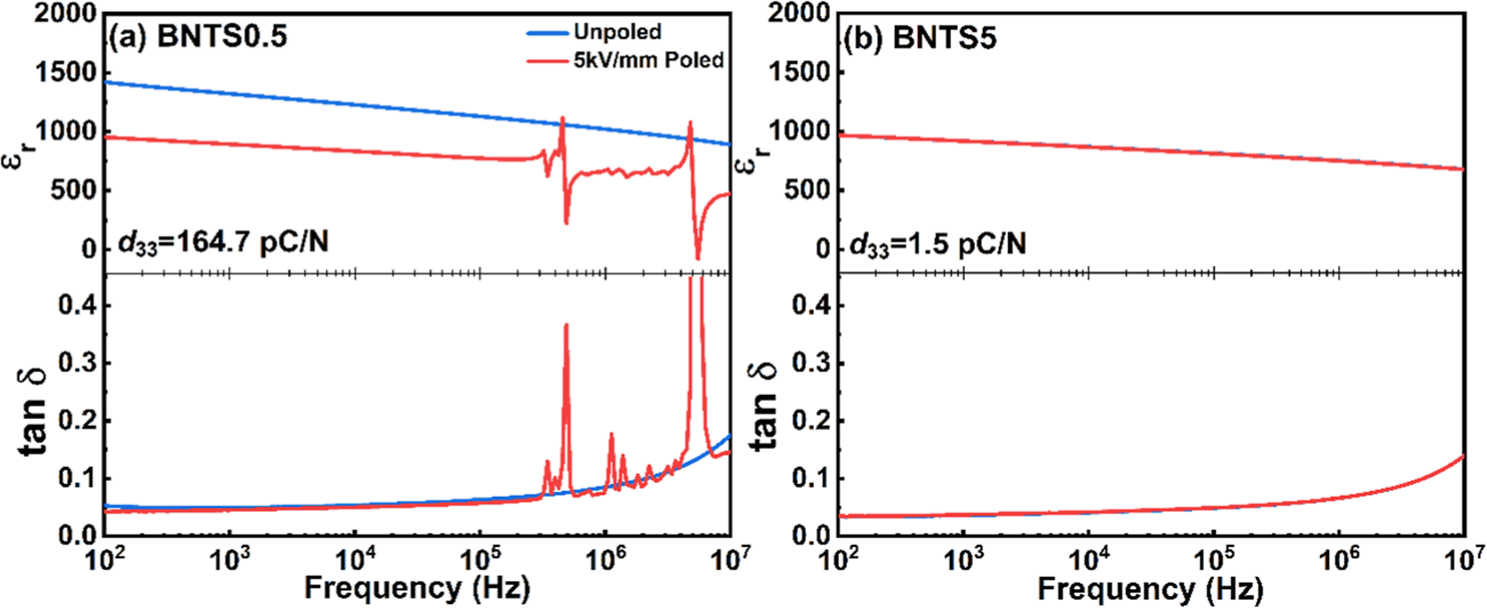
Frequency dependence of the dielectric permittivity (*ε_r_*) and loss (*tanδ*) for the unpoled and poled samples at room temperature: (a) BNTS0.5 and (b) BNTS5.

Figure 6 shows the frequency dependence of the relative dielectric permittivity (*ε_r_*) and loss (*tanδ*) of the unpoled and poled BNTS0.5 and BNTS5 ceramics at room temperature in the frequency range 100 Hz–10 MHz. Generally, the dielectric permittivity decreases with increasing frequency. This behavior is typical of ferroelectrics and can be attributed to the decreasing extrinsic contribution of domain walls to the permittivity as the excitation frequency of the applied field approaches the domain wall relaxation frequency (usually located in the GHz range).^42,44,45^ A comparison of the dielectric spectra of the unpoled and poled BNTS0.5 ceramics reveals that there is a significant decrease in the permittivity after poling, which is accompanied by a series of piezoelectric resonance peaks, both reflecting the nonergodic state of BNTS0.5. The permittivity decrease is thought to be caused by domain coalescence during DC poling, which leads to a reduction in domain wall density.^42^ On the other hand, the dielectric properties of the BNTS5 ceramic remain relatively unchanged after poling. This can be explained by the instability of the field-induced ferroelectric state in the originally ergodic BNTS5 ceramic and the recovery of the microstructural effects driven by the DC poling (e.g., domain structure changes) upon removal of the poling field. It is worth mentioning that after poling, the BNTS0.5 ceramic shows a relatively high piezoelectric *d*_33_ coefficient (∼164.7 pC N^-1^), if compared to the poled BNTS5 (∼1.5 pC N^-1^). Table S2 provides a summary of the dielectric, ferroelectric, and piezoelectric properties of BNTS0.5 and BNTS5 ceramics measured at room temperature.

By comparing the dielectric permittivities of BNTS0.5 and BNTS5 above *T*_s_ (Figure 4), one can see that BNTS5 has lower permittivity than BNTS0.5. The lower the permittivity, the higher the dielectric breakdown strength of a dielectric.^12^ The advantage of the higher breakdown strength of BNTS5 in high-power energy storage applications will be discussed with respect to an enhancement of the energy density in the following paragraphs.

The recoverable energy density is influenced by various parameters, including the remanent polarization, maximum polarization, and electrical breakdown strength. To maximize the breakdown strength at a given voltage, bulk ceramics are usually processed to small thicknesses by grinding. However, thin ceramics have a lower capacity for energy storage due to the reduced volume of material in which the energy can be stored. Moreover, in technical practice, applying large external electric fields often leads to high risk of electrical breakdown.^16,46^

In order to evaluate the energy storage performance of dielectrics, we have introduced the recoverable energy storage intensity (ρ), which represents the recoverable energy storage density under a certain electric field and can be expressed as:^16^

4 $$\rho =\frac{W_{rec}}{E}$$

where *E* is the applied electric field (always lower than the breakdown electric field). In our study, the *ρ* value of BNTS5 ceramics at room temperature was calculated to be 12.93 J V^–1^ cm^–2^. The *ρ* value of the BNTS5 at various temperatures is detailed in Table S3 (in the Supporting Information). Table 2 compares the energy storage properties of BNTS5 ceramics with those of other recently developed energy storage ceramics. Although some of the materials show higher *W*_rec_ than that of the BNTS5 ceramic, it is important to note that these higher values are due to higher applied electric fields. If one evaluates the energy storage performance according to the recoverable energy storage intensity, BNST5 shows the highest value of all of the reviewed ceramics (in Table 2) and can be regarded as an ideal candidate for energy storage at and close to room temperature.

**Table 2:** Energy Storage Properties of Ferroelectric Ceramics at Room Temperature

compounds^a^	*E* (kV cm^–1^)	*W*_rec_ (J cm^–3^)	*η* (%)	*ρ* (J V^–1^ cm^–2^)	ref
0.70BaTiO_3_-0.30BS	730	6.1	–^b^	8.36	^47^
BNBT-0.06KN	100	0.89	–	8.90	^48^
0.97(0.65BF-0.35BT)-0.03Nb_2_O_5_	90	0.71	–	7.89	^49^
0.70(0.94BNT-0.06BT)-0.30ST	90	0.98	82	10.89	^50^
0.88BaTiO_3_-0.12BMT	224	1.81	–	8.08	^51^
0.85BaTiO_3_-0.15BZT (MLCC)	330	2.8	–	8.48	^52^
0.91BaTiO_3_-0.09BY	93	0.71	82.6	7.63	^53^
0.90BaTiO_3_-0.10BMN	143.5	1.13	90	7.87	^54^
BBNT-0.15SZ	155	1.32	∼56	8.52	^55^
0.80(K_0.5_Na_0.5_)NbO_3_-0.20SSN	295	2.02	81.4	6.85	^56^
0.80KNN-0.20SSN-0.5%ZnO	400	2.6	73.2	6.50	^57^
0.85BaTiO_3_-0.15BZN	131	0.79	93.5	6.03	^58^
0.90(0.92BNT-0.08BT)-0.10NT	100	1.2	74.8	12.00	^59^
0.85(K_0.5_Na_0.5_)NbO_3_-0.15ST	400	4.03	52	10.08	^60^
0.80(K_0.5_Na_0.5_)NbO_3_-0.20ST	400	3.67	72.1	9.18	^60^
0.90LLBNTZ-0.10NBN	178	2.04	54.76	11.46	^61^
0.85BaTiO_3_-0.15BZS	280	2.21	91.6	7.89	^62^
BNT-Na	260	3.18	69.30	12.23	^16^
**BNTS5**	**300**	**3.88**	**71.06**	**12.93**	**This work**

a BT: BaTiO_3_; BS: BiScO_3_; BNBT: (Bi_0.47_Na_0.47_Ba_0.06_)­TiO_3_; KN: KNbO_3_; BF: BiFeO_3_; BNT: (Bi_0.5_Na_0.5_)­TiO_3_; ST: SrTiO_3_; BMT: Bi­(Mg_0.5_Ti_0.5_)­O_3_; BZT: Bi­(Zn_0.5_Ti_0.5_)­O_3_; BY: BiYbO_3_; BMN: Bi­(Mg_2/3_Nb_1/3_)­O_3_; BBNT: Ba_0.04_Bi_0.48_Na_0.48_TiO_3_; SZ: SrZrO_3_; KNN: (K_0.5_Na_0.5_)­NbO_3_; SSN: Sr­(Sc_0.5_Nb_0.5_)­O_3_; BZN: Bi­(Zn_2/3_Nb_1/3_)­O_3_; NT: NaTaO_3_; LLBNTZ: Bi_0.48_La_0.02_Na_0.48_Li_0.02_Ti_0.98_Zr_0.02_O_3_; NBN: Na_0.73_Bi_0.09_NbO_3_; BZS: Bi­(Zn_1/2_Sn_1/2_)­O_3_; BNT-Na: 0.94 Bi_0.5_Na_0.4202_Sm_0.0266_TiO_3_ −0.06BaTiO_3_.

b “–”: data not provided.

The value of dielectric breakdown strength (*E*_bs_) was obtained by the Weibull analysis using:^63,64^

5 $$X_{i}=\ln\left(E_{i}\right)$$

6 $$Y_{i}=\ln\left[-\ln\left(1-P_{i}\right)\right]$$

7 $$P_{i}=\frac{i}{1+n}$$

where *X*_*i*_ and *Y*_*i*_ are the variables of the Weibull distribution function, *E*_*i*_ is the specific breakdown field of the *i*th samples arranged in an ascending order, *P*_*i*_ is the probability for dielectric breakdown, and *n* is the total number of samples (*n* = 10).

As can be seen in Figure 7a, there is a linear relationship between *X*_i_ and *Y*_i_, with a slope *β* (the so-called Weibull modulus) of about 17. It is generally accepted that a Weibull analysis with *β* ≥ 10 indicates high reliability.^65,66^ The higher the value of *β*, the smaller the scattering range of the tested *E*_bs_ values. In our work, a characteristic strength *E*_bs_ value of about 30.6 kV/mm was obtained from the linear fit for *Y*_i_ = 0.

**Figure 7:**
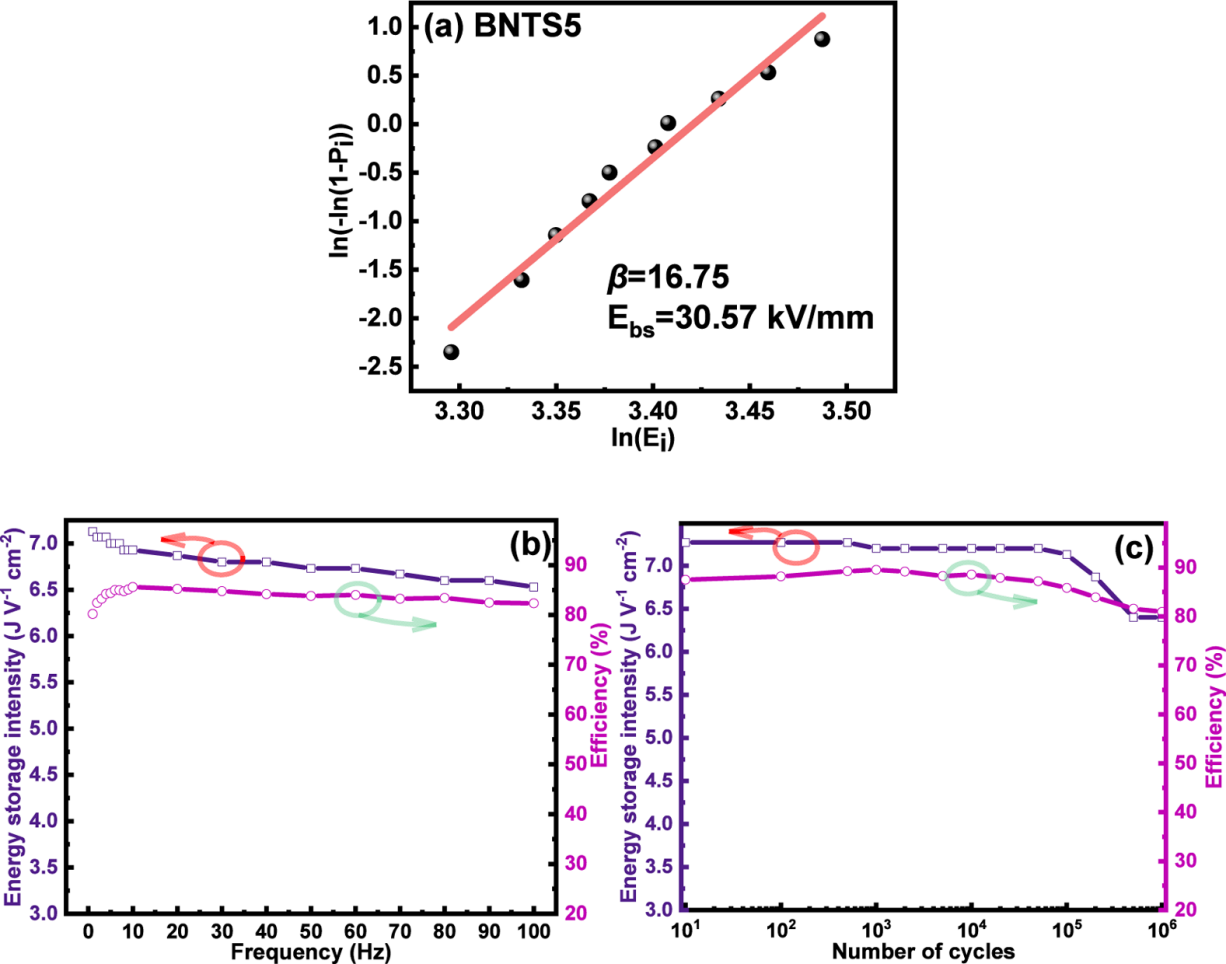
(a) The Weibull distribution of the *E*_bs_ for the BNTS5 ceramics; (b) variation in the energy storage performance of BNTS5 ceramics with frequency at room temperature; and (c) variation in the energy storage performance of BNTS5 as a function of the number of cycles at room temperature.

For practical applications of dielectric capacitors, both frequency and cycling stability are important characteristics. As shown in Figures 7b and S6 (Supporting Information), the BNTS5 ceramic exhibits an excellent frequency stability over a wide frequency range (1–100 Hz). Moreover, it demonstrates a remarkable cycling stability, with stable performance from 10 cycles to 10^6^ cycles, as shown in Figures 7c and S7 (Supporting Information). Considering that capacitors are typically operated at 33–40% of their breakdown strength to ensure a reliable performance under practical conditions,^64^ an electric field of 15 kV mm^–1^, which is approximately 50% of the breakdown field (*E*_bs_ ∼ 30.6 kV/mm) of the BNTS5 ceramic, was applied during cycling and temperature stability tests. The energy storage performance of BNTS5 ceramics at various frequencies and after numerous switching cycles is given in Tables S4 and S5 (Supporting Information), respectively.

## 4 Conclusions

The Sm-doped BNT-6BT ceramics, namely, BNTS0.5 and BNTS5, were prepared by the conventional solid-state reaction method. The incorporation of the Sm ions into the A-site of the BNT perovskite modifies the atomic arrangement on a nanometer scale, resulting in changes of the phase transition temperatures, decreasing the temperature *T*_s_ and increasing *T*_m_ upon doping. The BNTS0.5 sample was found to show a relatively high piezoelectric *d*_33_ coefficient (∼164.7 pC N^-1^) at room temperature, making the ceramic a good material for piezoelectric applications. Moreover, the presence of eight peaks in the current–electric field loops was attributed to a multistage field-induced transition process. The highly doped BNTS5 showed excellent energy storage properties at room temperature, with a recoverable energy storage intensity *ρ* = 12.93 J V^–1^ cm^–2^, recoverable energy storage density *W*_rec_ = 3.880 J cm^–3^, and storage efficiency *η* = 71.06%. These values classify the BNTS5 ceramic as a superior candidate for room-temperature energy storage applications. When the two ceramics are compared, BNTS5 exhibits a high dielectric breakdown strength related to its low permittivity. BNTS0.5 shows high energy storage performance only at high temperatures due to large remnant polarization at room temperature. In contrast, BNTS5 performs well at room temperature due to a stable ergodic state, higher *P*4*bm* phase content, reduced remnant polarization, and enhanced dielectric breakdown strength from the low permittivity.

The possibility of modifying the temperatures *T*_s_ and *T*_m_, the dielectric and piezoelectric properties, as well as the electric field-induced phase transitions through appropriate compositional variations and nanoscale engineering enhances the versatility of BNT-based ceramics. In this context, the Sm doping approach offers an additional layer of tunability to specific properties, supporting the development of novel lead-free relaxor ferroelectrics for emerging piezoelectric energy conversion and energy storage applications.

## Supplementary Material



## Data Availability

The data supporting this article have been included as part of the Supporting Information.

## Notes

Sintered densities for both ceramic compositions; SEM–EDX elemental mapping of the BNTS0.5 and BNTS5 ceramics; *P*–*I*–*E* hysteresis loops of both materials (at various temperatures and electric fields); characteristic dielectric, ferroelectric, and piezoelectric parameters of the Sm-doped ceramics; energy storage density, efficiency, and intensity of the BNTS5 ceramic; and *P*–*E* hysteresis loops of the BNTS5 ceramic at different frequencies and after several switching cycles (PDF)
