# Supplementary material for: Effect of Samarium Doping on the Energy Storage Properties of Bismuth Sodium Titanate-Based Lead-Free Ceramics
Source: ACS Appl Mater Interfaces. 2025 Sep 10;17(38):53780–90. doi: 10.1021/acsami.5c12016 (PMC12464909; doi:10.1021/acsami.5c12016)
Supplement: Supplementary file 1 [file am5c12016_si_001.pdf]

# Supporting Information

# Effect of samarium doping on the energy storage properties of bismuth sodium titanate-based lead-free ceramics

Xuyao Tang[a], Wanting Hu[a], Vladimir Koval[b]*, Jiangtao Zeng[c], Giuseppe Viola[a], Haixue Yan[a]*

[a]*School of Engineering and Materials Science, Queen Mary University of London, Mile End Road, London E1 4NS, UK*

[b]*Institute of Materials Research, Slovak Academy of Sciences, 04001 Kosice, Slovakia*

[c]*Shanghai Key Laboratory of Engineering Materials Application and Evaluation, Shanghai Research Institute of Materials, Shanghai 200437, P. R. China*

*E-mail address:* xuyao.tang@qmul.ac.uk (Xuyao Tang), w.hu@qmul.ac.uk (Wanting Hu), vkoval@saske.sk (Vladimir Koval), zjt21cn@163.com (Jiangtao Zeng), g.viola@qmul.ac.uk (Giuseppe Viola), h.x.yan@qmul.ac.uk (Haixue Yan).

* *Corresponding authors.*

*E-mail: vkoval@saske.sk (*V. Koval*), h.x.yan@qmul.ac.uk (*H. Yan*).*

**Table S1.** The sintered density, as obtained by the Archimedes' method, and the corresponding relative density of the BNTS0.5 and BNTS5 ceramics.

| Sample | Density ($g/cm^3$) | Theoretical Density | Relative Density |
|---|---|---|---|
| BNTS0.5 | 5.83 ± 0.02 | 5.95 | 97.98 ± 0.34 % |
| BNTS5 | 5.88 ± 0.02 | 6.01 | 97.84 ± 0.33 % |

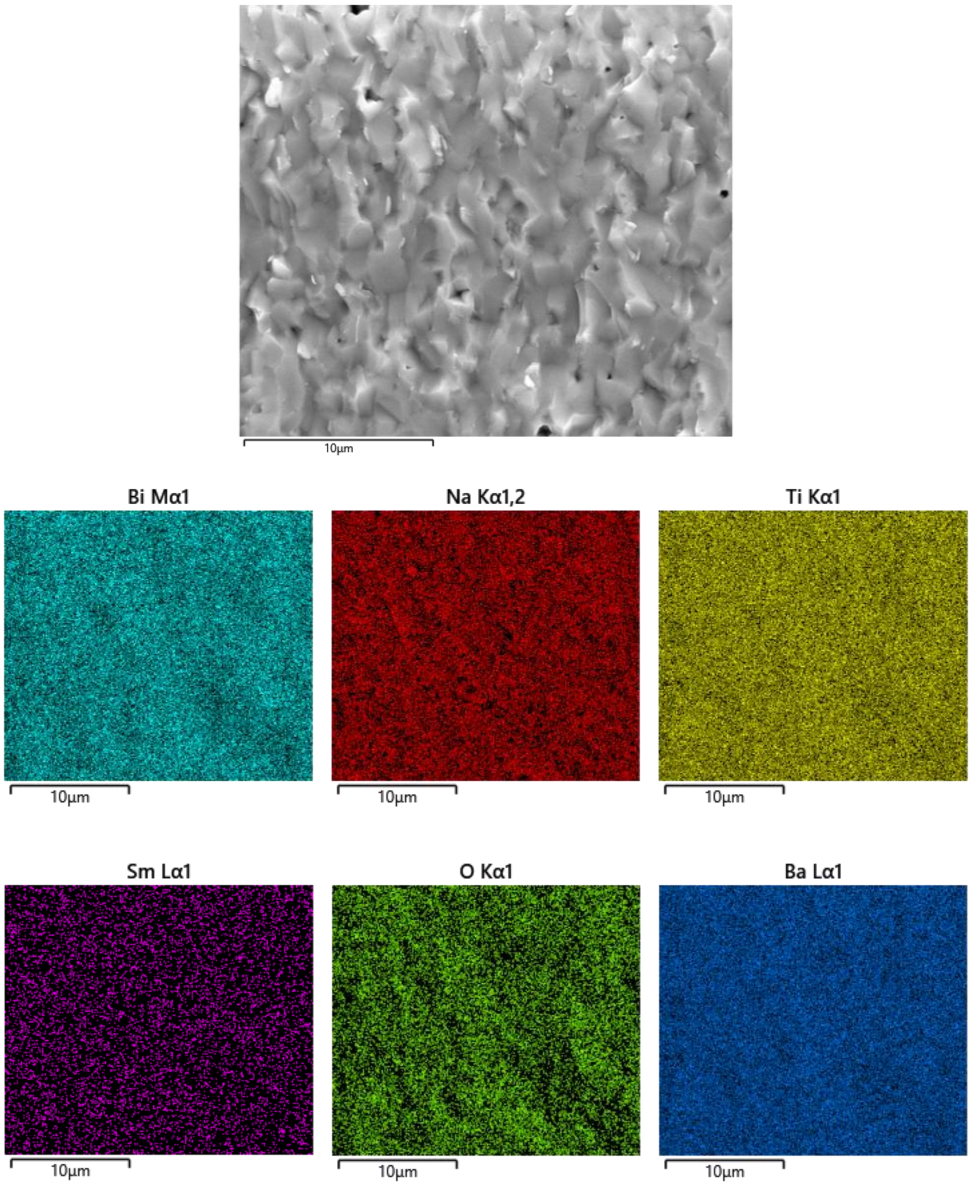


**Figure S1.** EDX elemental mapping of the BNTS0.5 ceramic.

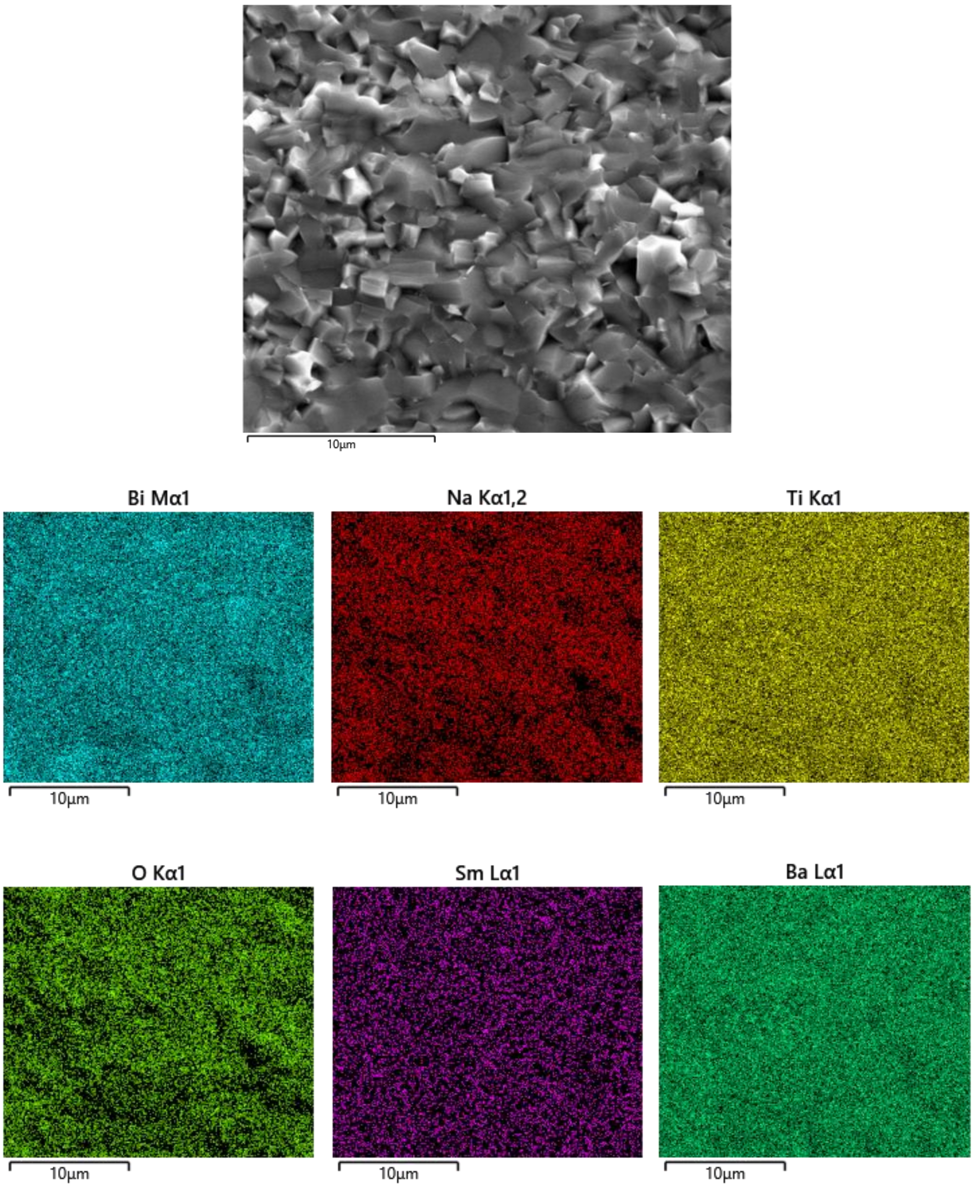


**Figure S2.** EDX elemental mapping of the BNTS5 ceramic.

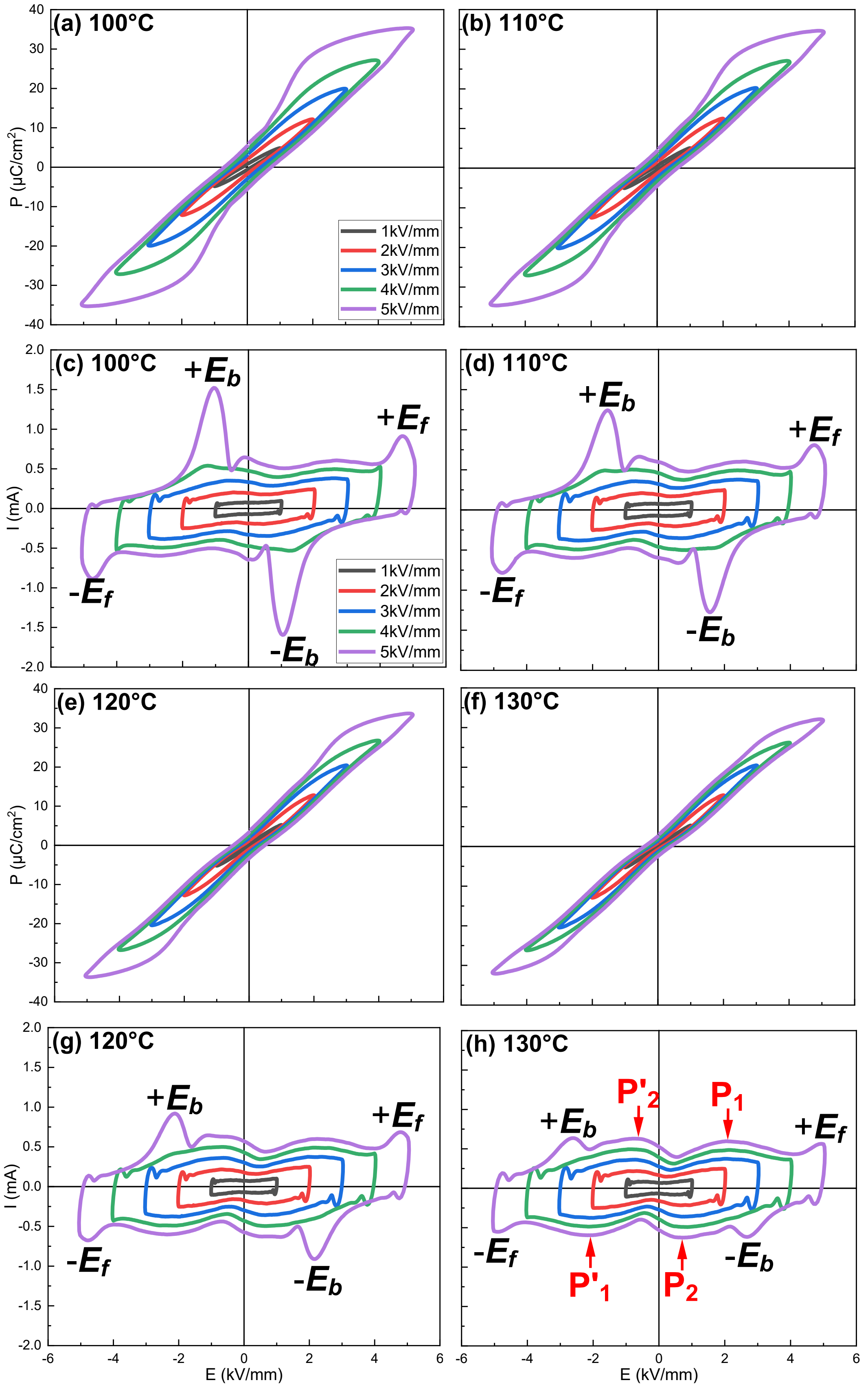


**Figure S3.** *P-E* (a, b, e, f) and *I-E* (c, d, g, h) loops of BNTS0.5 ceramics, as recorded at selected temperatures over the range 100°C - 130°C under an electric field of 1, 2, 3, 4 and 5 kV $mm^{-1}$.

**Table S2.** The dielectric, ferroelectric and piezoelectric properties of the BNTS0.5 and BNTS5 ceramics at room temperature.

| **Sample** | **Dielectric permittivity (1 kHz)** | **Dielectric loss (1 kHz)** | $d_{33}$ **(pC N$^{-1}$)** | $T_s$ **(°C)** | $T_m$ **(°C)** | $P_r$ **(μC cm$^{-2}$)** |
|---|---|---|---|---|---|---|
| BNTS0.5 | 1319.97 | 0.049 | ±164.7 | 122 | 265 | 28.01 |
| BNTS5 | 916.86 | 0.036 | ±1.5 | 44 | 304 | 3.31 |

Notes: $d_{33}$ - piezoelectric coefficient, $T_\mathrm{s}$ - depolarization temperature, $T_\mathrm{m}$ -temperature of the maximum permittivity, $P_\mathrm{r}$ - remanent polarization.

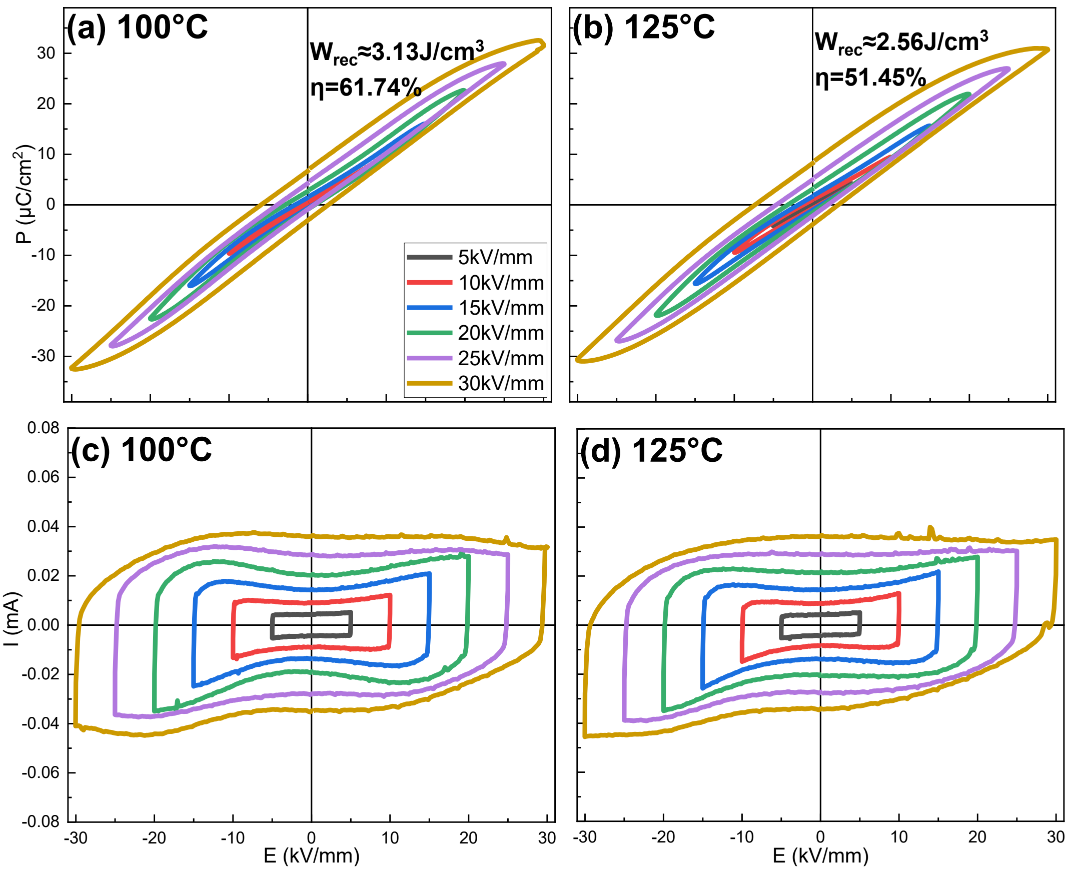


**Figure S4.** *P-E* and *I-E* loops of BNTS5 ceramics, as measured at a temperature of: (a,c) 100 °C and (b,d) 125 °C under an electric field of 5, 10, 15, 20, 25 and 30 kV $mm^{-1}$.

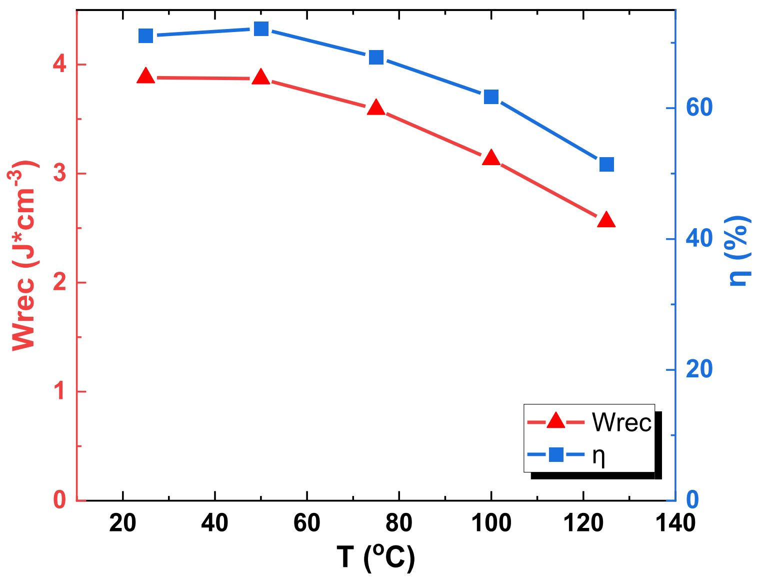


**Figure S5.** The recoverable energy storage density ($W_{rec}$) and energy storage efficiency ($\eta$) as function of temperature for BNTS5 (at 30 kV $mm^{-1}$).

**Table S3.** Energy storage properties of the BNTS5 ceramic at various temperatures.

| **Temperature (°C)** | $E$ **(kV cm$^{-1}$)** | $W_{rec}$ **(J cm$^{-3}$)** | $\eta$ **(%)** | $\rho$ **(J V$^{-1}$ cm$^{-2}$)** |
|---|---|---|---|---|
| 25 | 300 | 3.88 | 71.06 | 12.93 |
| 50 | | 3.87 | 72.15 | 12.90 |
| 75 | | 3.59 | 67.81 | 11.97 |
| 100 | | 3.13 | 61.74 | 10.43 |
| 125 | | 2.56 | 51.45 | 8.53 |

Notes: $E$ - the applied field (always lower than the breakdown electric field), $W_{rec}$ - recoverable energy storage density, $\eta$ - energy storage efficiency, $\rho$ - recoverable energy storage intensity.

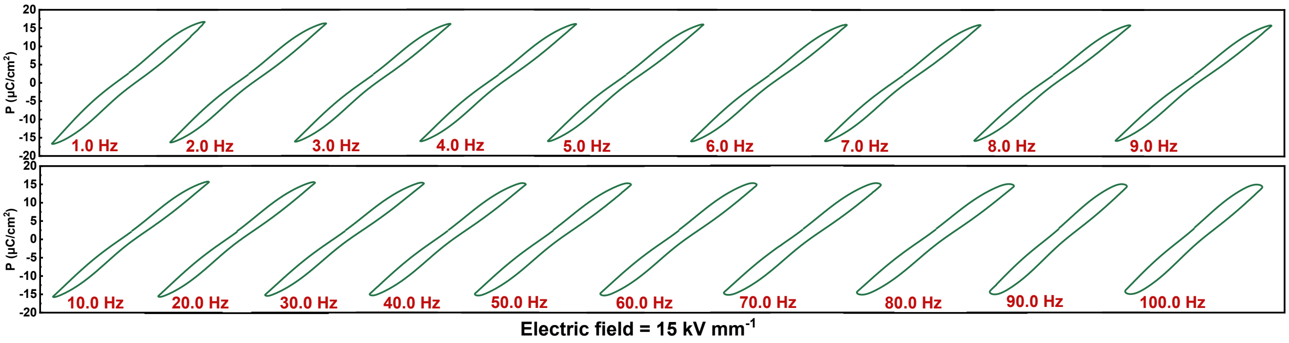


**Figure S6.** *P-E* loops at different frequencies for the BNTS5 ceramics at room temperature.

**Table S4.** Energy storage properties of the BNTS5 ceramic at various frequencies.

| **Frequency (Hz)** | ***E* (kV cm$^{-1}$)** | **$W_{rec}$ (J cm$^{-3}$)** | **$\eta$ (%)** | **$\rho$ (J V$^{-1}$ cm$^{-2}$)** |
|---|---|---|---|---|
| 1 | 150 | 1.07 | 80.21 | 7.13 |
| 2 | | 1.06 | 82.44 | 7.07 |
| 3 | | 1.06 | 83.24 | 7.07 |
| 4 | | 1.06 | 84.23 | 7.07 |
| 5 | | 1.05 | 84.40 | 7.00 |
| 6 | | 1.05 | 85.01 | 7.00 |
| 7 | | 1.05 | 84.99 | 7.00 |
| 8 | | 1.04 | 84.76 | 6.93 |
| 9 | | 1.04 | 85.12 | 6.93 |
| 10 | | 1.04 | 85.62 | 6.93 |
| 20 | | 1.03 | 85.23 | 6.87 |
| 30 | | 1.02 | 84.79 | 6.80 |
| 40 | | 1.02 | 84.18 | 6.80 |
| 50 | | 1.01 | 83.82 | 6.73 |
| 60 | | 1.01 | 84.02 | 6.73 |
| 70 | | 1.00 | 83.26 | 6.67 |
| 80 | | 0.99 | 83.43 | 6.60 |
| 90 | | 0.99 | 82.47 | 6.60 |
| 100 | | 0.98 | 82.35 | 6.53 |

Notes: $E$ - the applied field (always lower than the breakdown electric field), $W_{rec}$ - recoverable energy storage density, $\eta$ - energy storage efficiency, $\rho$ - recoverable energy storage intensity.

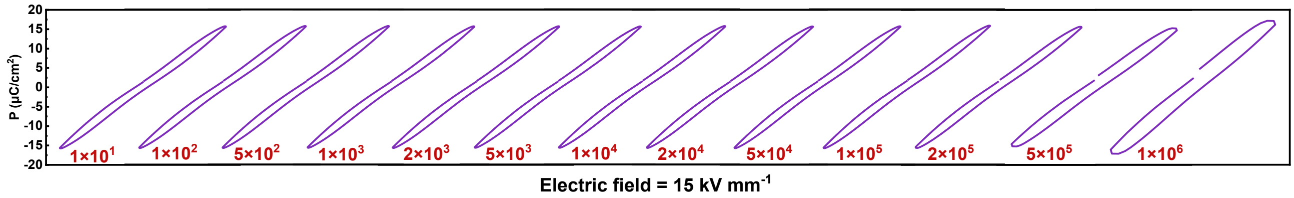


**Figure S7.** *P-E* loops recorded at room temperature after a number of switching cycles for the BNTS5 ceramic at room temperature.

**Table S5.** Number of cycles and energy storage properties of the BNTS5 ceramic.

| **Number of cycles** | ***E*** **(kV cm$^{-1}$)** | **$W_{rec}$ (J cm$^{-3}$)** | **$\eta$ (%)** | **$\rho$ (J V$^{-1}$ cm$^{-2}$)** |
|---|---|---|---|---|
| $1\times10^1$ | 150 | 1.09 | 87.50 | 7.27 |
| $1\times10^2$ | | 1.09 | 88.19 | 7.27 |
| $5\times10^2$ | | 1.09 | 89.26 | 7.27 |
| $1\times10^3$ | | 1.08 | 89.55 | 7.20 |
| $2\times10^3$ | | 1.08 | 89.18 | 7.20 |
| $5\times10^3$ | | 1.08 | 88.27 | 7.20 |
| $1\times10^4$ | | 1.08 | 88.56 | 7.20 |
| $2\times10^4$ | | 1.08 | 87.89 | 7.20 |
| $5\times10^4$ | | 1.08 | 87.19 | 7.20 |
| $1\times10^5$ | | 1.07 | 85.86 | 7.13 |
| $2\times10^5$ | | 1.03 | 83.94 | 6.87 |
| $5\times10^5$ | | 0.96 | 81.58 | 6.40 |
| $1\times10^6$ | | 0.96 | 80.95 | 6.40 |

Notes: $E$ - the applied field (below the breakdown electric field), $W_{rec}$ - recoverable energy storage density, $\eta$ - energy storage efficiency, $\rho$ - recoverable energy storage intensity.
